# Benzoxanthenone Lignans Related to Carpanone, Polemanone, and Sauchinone: Natural Origin, Chemical Syntheses, and Pharmacological Properties

**DOI:** 10.3390/molecules30081696

**Published:** 2025-04-10

**Authors:** Christian Bailly

**Affiliations:** 1 UMR9020-U1277-CANTHER—Cancer Heterogeneity Plasticity and Resistance to Therapies, CHU Lille, CNRS, Inserm, OncoLille Institut, University of Lille, 59000 Lille, France; christian.bailly@univ-lille.fr; 2 Institute of Pharmaceutical Chemistry Albert Lespagnol (ICPAL), Faculty of Pharmacy, University of Lille, 59006 Lille, France; 3 OncoWitan, Scientific Consulting Office, 59290 Lille, France

**Keywords:** *Saururus chinensis*, benzoxanthenones, sauchinone, carpanone, polemannones, biological activities, anticancer, anti-inflammatory, antioxidant

## Abstract

Medicinal plants from the genus *Saururus* are commonly used to treat inflammatory pathologies. They contain numerous bioactive compounds, notably the polycyclic lignan sauchinone from the species *Saururus chinensis*. An in-depth analysis of benzoxanthenone lignans related to sauchinone, and the analogous products carpanone and polemannones, has been carried out. The review reports the product’s isolation, biosynthetic pathway, and chemical strategies to synthesize benzoxanthenones via liquid- and solid-phase syntheses. The metabolic and pharmacokinetic properties of sauchinone are discussed. At the pharmacological level, sauchinone is a potent blocker of the production of pro-inflammatory mediators, such as nitric oxide and prostaglandin E2, and an efficient antioxidant agent. The properties of sauchinone can be exploited to combat multiple pathologies, such as liver injuries, renal dysfunction, osteoarthritis, inflammatory bowel disease, ulcerative colitis, and cancers. The capacity of the natural product to inhibit tumor cell proliferation and to reduce migration/invasion of cancer cells and the development of metastases is underlined, together with the regulation of the epithelial-mesenchymal transition and immune checkpoints. Altogether, the review offers a complete survey of the chemical and biochemical properties of sauchinone-type benzoxanthenones.

## 1. Introduction

Plants from the genus *Saururus* are commonly used in traditional medicine for the treatment of multiple health conditions and inflammatory pathologies [1]. The genus comprises two species, *Saururus cernuus* L. and *Saururus chinensis* (Lour) Baill. The former is native to the temperate zones of eastern North America, and the latter comes from East Asia essentially. Both belong to the Saururaceae, a very small family in the order Piperales within the magnoliid clade [1]. These plants exist for a very long time. An analysis of the pollen morphology in Saururaceae has revealed a highly conservative structure, which has remained largely unchanged for the last 80 million years. Dispersed fossil pollen grains have been dated from the Campanian (82–81 Ma) [2].

**Figure 1:**
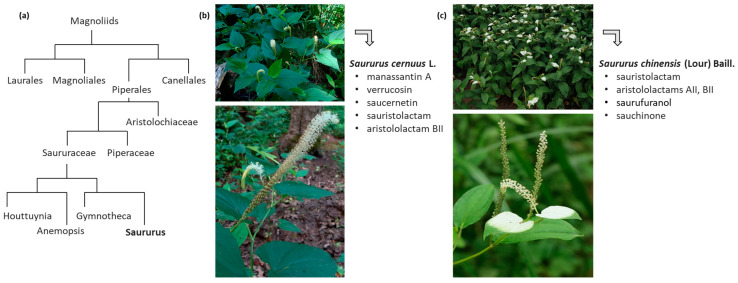
(**a**) Composition and phylogeny of the magnoliid order Piperales, with the position of the *Saururus* genus. (**b**) The plant *Saururus cernuus* L. (from Arkansas Native Plant Society, (https://anps.org/2015/07/08/know-your-natives-lizards-tail/, accessed 18 March 2025)). (**c**) *Saururus chinensis* (Lour) Baill. (from Plant of the World Online (POWO, https://powo.science.kew.org/taxon/urn:lsid:ipni.org:names:684080-1, accessed 18 March 2025)).

The name of *Saururus* plants derives from the Latin word saurus, which means lizard. The flower of *Saururus* resembles a lizard’s tail. For this reason, the two species are often referred to as the lizard’s tail family, with the species *S. chinensis* known as Asian lizard’s tail (Sam-baekcho in Korea). The two plants display perianthless flowers and petaloid bracts, but no petals or sepals [3,4]. The inflorescences are elongated, up to 6–8 inches long (Figure 1). The epithet *cernuus* means nodding, with reference to the appearance of the tail-like nodding inflorescence. These perennial, herbaceous plants are generally found in wetlands with light shade to partial sun. They appreciate permanently or periodically flooded habitats, such as swamps, where they can form dense colonies.

*Saururus* species are important medicinal plants utilized to treat various human pathologies. An ancient study published in Atlanta (USA) in 1867 referred to the use of the whole plant *S. cernuus* to ally pain and to prevent suppuration in mammary inflammation [5]. Extracts from this species have shown antiparasitic and anticancer activities [6]. Various bioactive natural products have been isolated from *S. cernuus*, in particular, the neolignan manassantin A endowed with marked anticancer properties via the targeting of Hsp90 chaperone, the mitochondrial complex I, and other protein targets [7,8,9]. Diverse other neolignans have been isolated from *S. cernuus*, such as verrucosin and saucernetin, endowed with cytotoxic and/or antiparasitic properties [10,11,12]. A few alkaloids have been found also in *S. cernuus*, such as sauristolactam and aristololactam BII, also known as cepharanone B [13].

**Figure 2:**
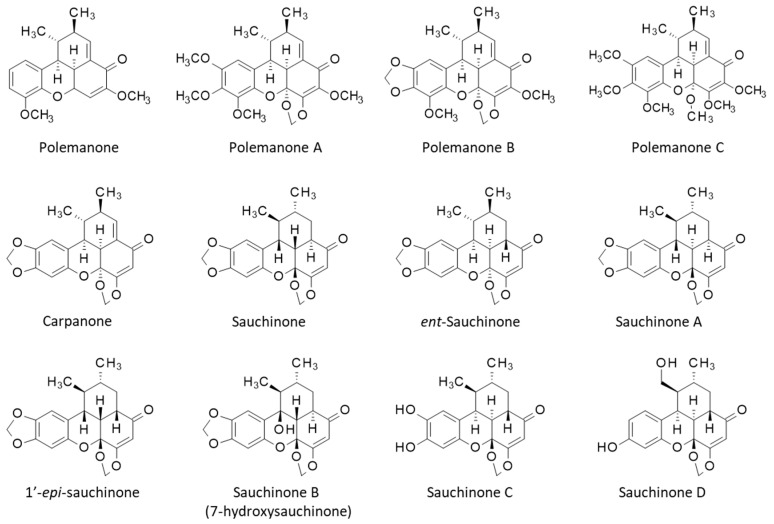
Structures of carpanone, polemannones, and sauchinones.

Similar alkaloids have been isolated from the Asian species *S. chinensis*, notably aristolactams AII, BII, and sauristolactam, in addition to nitroalkyl indole alkaloids [14,15,16]. However, the alkaloid content is limited. Diverse common flavonoids, anthraquinone derivatives, saponins, and phenols have been identified also. But the plant is essentially known for the presence of numerous lignans. In 2020, Liu and coworkers repertoried >100 lignans isolated from the two *Saururus* species, including dibenzylbutanes, benzofurans, tetrahydrofurans, arylnaphthalenes, and other type of lignans [1]. New compounds are regularly identified, such as the furanoditerpene compound named saurufuranol, isolated very recently [17]. Many of these lignans have revealed anti-inflammatory and/or antitumor activities. Among them, the polycyclic lignan sauchinone (Figure 2), initially isolated from *S. chinensis*, has emerged as a lead natural product of interest to combat diverse human pathologies, including cancers and inflammatory diseases.

An in-depth analysis of sauchinone-type lignans has been carried out with the objectives to highlight the interest of these natural products and to promote research in this field. A focus was made on lignans bearing a benzoxanthenone core, including mainly carpanone, polemannones, sauchinones, and derivatives.

## 2. Isolation and Biosynthesis of Sauchinone and Related Lignans

Sauchinone was isolated for the first time from a total extract of *S. chinensis* 30 years ago [18]. It was followed with the identification of the diastereoisomers designated sauchinone A and 1′-*epi*-sauchinone) [19], 7-hydroxysauchinone (sauchinone B), the two analogues sauchinones C-D [20,21], and then the neolignan *ent*-sauchinone [22]. These compounds are considered as markers for *S. chinensis*. Sauchinone has been rarely identified in other plants. However, its presence has been mentioned recently in a totally distinct medicinal plant, *Choerospondias axillaris*, based on a mass spectrometry analysis [23].

The sauchinone content in the aerial part of *S. chinensis* is relatively high, in the range 0.67–4.05 mg/g of dried plant. The compound tends to accumulate in the plant during the fruiting period, between July and August, but the seasonal variation is not large. The compound remains present in the plant all along the year, mostly in the aerial part of the plant [24,25,26,27]. However, the compound has been isolated from *S. chinensis* roots in some cases [28,29]. Sauchinone derivatives have been found in the roots, such as 5′α- and 5′β-hydroxy-4′-methoxy-sauchinone isolated together with the parent compound and other molecules (e.g., machilin A, saucerneol C, macelignan, (−)-chicanin, and a few others) [30].

**Scheme 1:**
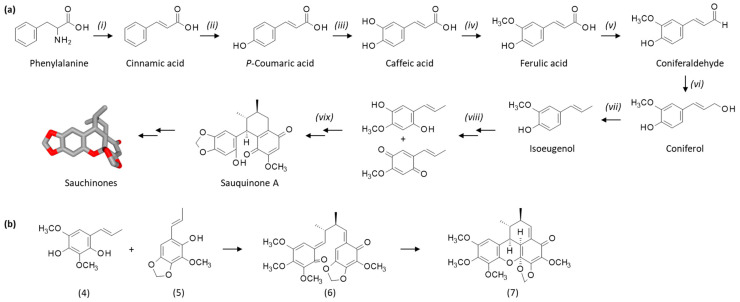
(**a**) Biosynthetic pathway to sauchinone-type natural products. The phenylalanine precursor is successively transformed into isoeugenol, as a key precursor to the various (*ent*- or *epi*-)-sauchinone derivatives. Compound sauquinone A is one of the identified intermediates [22,31,32]. (i) phenylalanine ammonia-lyase, (ii) cinnamate-4-hydroxylase, (iii) *p*-coumarate-3-hydroxylase, (iv) caffeic acid 3-*O*-methyl transferase, (v) cinnamoyl-CoA reductase, (vi) cinnamyl-alcohol dehydrogenase, (vii) oxidative coupling, (ix) coniferyl alcohol acyltransferase and isoeugenol synthase 1, and (viii) β,β-phenolic coupling Diels–Alder reaction. (**b**) Biosynthetic pathway to polemannone-type natural products. Compound (**7**) (4,5-dimethoxy-4′,5′-methylenedioxypolemannone) would be formed biogenetically by phenolic oxidation of (**4**) followed by reaction with (**5**) leading to (**6**) via a heterodien reaction. Polemannone derivative (**7**) has been isolated together with two analogues: 4,5,4′,5′-bismethylenedioxypolemannone and 4,5,4′,5′-tetramethoxypolemannone [33].

The biosynthetic pathway leading to sauchinone has been delineated, at least partially. The precursor is the amino acid phenylalanine, which is converted to intermediates like coniferaldehyde and isoeugenol. This latter phenylpropanoid compound is a key precursor for many small molecules. The transformation process from isoeugenol to sauchinone has not been firmly established, but a hypothetical biosynthetic pathway has been proposed, as illustrated in Scheme 1 [22]. A recent transcriptome analysis identified 28 candidate genes encoding 12 enzymes involved in the biosynthesis pathway leading to sauchinone and related lignans [31]. One of the precursors of sauchinone would be a benzoquinone derivative related to the product designated sauquinone A, identified from *S. chinensis* in 2019 [32] (Scheme 1a). A slightly distinct process has been described for the biosynthesis of the related products polemannones, initially isolated from *Polemannia* species. The authors proposed a biosynthetic pathway for the derivative 4,5-dimethoxy-4′,5′-methylenedioxypolemannone (**7**) from the phenolic oxidation of the precursor of (**4**) followed by reaction with (**5**) leading to (**6**). Cyclization of the compound (**6**) afforded (**7**) via a heterodien reaction, as shown in Scheme 1b [33].

Different natural products with a pentacyclic scaffold similar to that of sauchinone have been isolated, in particular those designated polemannones A–C and carpanone from the *Carpano* tree [34] (Figure 2). The biosynthetic route leading to these compounds has not been described, but the molecules have attracted considerable interest in medicinal chemistry.

## 3. Chemical Syntheses of Carpanone, Polemannone, and Sauchinone 

**Scheme 2:**
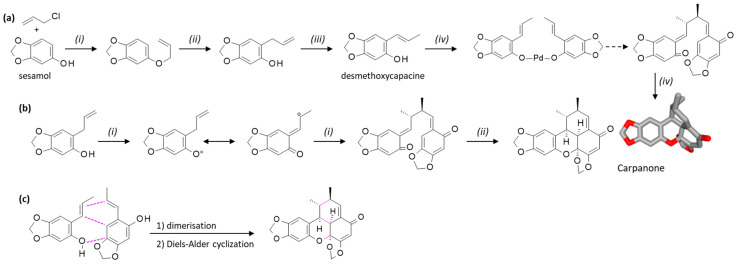
(**a**) Total synthesis of carpanone initially proposed [35,36]. (i) Reaction of sesamol with allyl chloride in the presence of KOH in ethanol at reflux. (ii) Claisen rearrangement at 220–270 °C. (iii) Isomerization of the double bond of 2-allyl-4,4-methylenedioxyphenol with potassium *tert*-butoxyde (KOC(CH_3_)_3_) in DMSO. (iv) Dimerization of *trans*-2-(1-propenyl)-4,5-methylenedioxyphenol in the presence of PdCl_2_ and Na acetate in aqueous methanol (MeOH/CH_3_OH 5:1) for 2 h at 38°C (46% yield). (**b**) Synthesis of carpanone in the presence of a transition metal(II) Schiff base and molecular oxygen [37]. (i) Oxidation of *trans*-2-(1-propenyl)-4,5-methylenedioxyphenol in the presence of O_2_ and the salen-Co(II) complex bis(salicylidene)-ethylenediamine in CH_2_Cl_2_ for 1.5 h at room temperature (rt) selectively affords the bis-*O*-quinonemethide-*trans* derivative (not the *cis*). (ii) Then, an intramolecular cycloaddition leads to carpanone. (**c**) Synthesis of carpanone-type benzoxanthenones via oxidative dimerization of an alkenyl phenol derivative. The starting phenol derivative was converted into the hexacyclic product in the presence of a vanadium(V) catalyst (20 mol %) and AgCl (2 equiv.) for 18 h at 40°C and ambient light in 1,2-dichloroethane (0.2 M) [38].

The natural products carpanone, polemannone, and sauchinone present the same tetracyclic benzoxanthenone scaffold, which can be obtained from the oxidation of an alkenyl phenol monomer in the presence of palladium (II), as represented in Scheme 2a. The initial synthesis of carpanone from sesamol, first proposed > 50 years ago, has been optimized in recent years [35,36]. Matsumoto and Kuroda [37] proposed a process based on the oxidation of *trans*-2-(1-propenyl)-4,5-methylenedioxyphenol in the presence of oxygen and a salen-based transition metal complex, such as Co(II)salen, Co(II)salpr, Fe(II)salen, and Mn(II)salen. The best yield (94%) was obtained with the Co(II)salen complex. The reaction process is depicted in Scheme 2b. Another efficient procedure has been proposed recently for the oxidative dimerization of alkenyl phenol derivatives in the presence of a vanadium catalyst and AgCl, leading to various benzoxanthenones [38] (Scheme 2c).

**Scheme 3:**
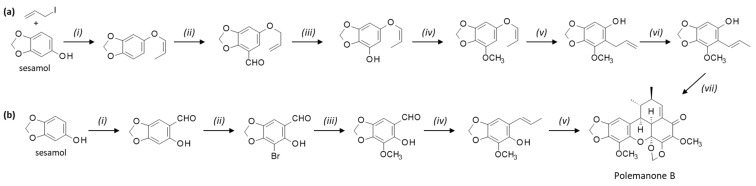
(**a**) Total syntheses of polemannone B from sesamol. (**a**) Automated synthesis using a reconfigurable flow platform. (i) Allylation of sesamol with allyl iodine and KOH in MeOH/H_2_O. (ii) Formylation with DMF/*n*-BuLi. (iii) Baeyer–Villiger oxidation of the aldehyde with *m*-chloroperbenzoic acid (*m*-CPBA) in CHCl_3_ at 0 °C. (iv) Etherification with methyl iodide (MeI/K_2_CO_3_) in acetone. (v) [3-3]-Claisen rearrangement in acetone at 250 °C and 70 bar. (vi) Isomerization by treatment with *t*-BuOK. (vii) Oxidative dimerization to obtain polemannone B [40]. (**b**) Cu(II)/sparteine-based coupling. (i) Formylation of sesamol in the presence of dichlorodimethyl ether and tin chloride (SnCl_2_) in CH_2_Cl_2_ for 3 h at room temperature (rt). (ii) Bromination in the presence of AlCl_3_ and Br_2_ in CH_2_Cl_2_ for 12 h at rt. (iii) Etherification in the presence of CuCl_2_, NaOMe in MoOH/DMF for 32 h at reflux. (iv) E-selective Wittig reaction to produce the styrenyl phenol derivative in the presence of Ph_3_PCH_2_CH_2_Br and *n*-BuLi in THF for 3 h at rt. (v) Treatment with the catalytic Cu(II)/sparteine oxidation system to obtain polemannone B. A similar process can be used to synthesize polemannone C [47].

A flexible platform has been designed for the optimized synthesis of carpanone using a modular autonomous flow reactor. The platform allowed the synthesis of carpanone in four steps with 67% overall yield from the precursor desmethoxycapacine obtained from sesamol [39]. Subsequently, the authors refined the procedure to optimize the dimerization of 2-propenyl phenols using a cobalt-catalyzed oxidizing system. The procedure led to the efficient synthesis of carpanone, polemannone B, and analogues [40] (Scheme 3a). Other approaches have been reported for the synthesis of carpanone and synthetic derivatives, generally starting from sesamol or styrenyl phenol precursors [41,42,43]. Of note, the use of a laccase enzyme for the stereo-controlled oxidative dimerization of the phenol precursor ((*E*)-2-propenylsesamol) has been proposed [44,45,46]. Similarly, synthetic routes have been proposed to prepare polemannone analogues. For example, the total synthesis of polemannones B and C from commercial starting materials has been accomplished with overall yields of 15% and 31.5%, respectively [47] (Scheme 3b). Efficient procedures have been reported also for carpanone and synthetic derivatives [42,43].

**Figure 3:**
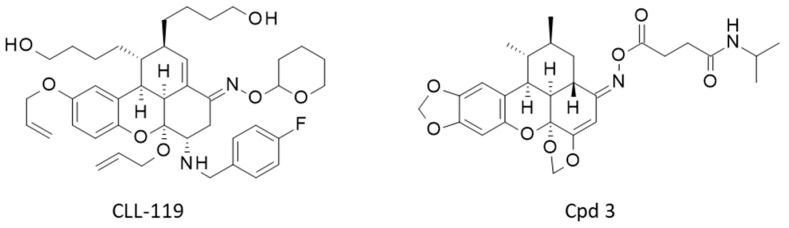
Structures of CLL-119 and a *ent*-sauchinone amide derivative (cpd 3) [50,52].

A polymer-based synthesis of carpanone has been implemented twenty years ago [48,49]. Subsequently, the solid-phase synthesis was refined to allow the preparation of a 10,000-membered library of molecules based on the carpanone core structure. This library was used to screen for inhibitors of exocytosis from the Golgi apparatus. A few compounds that inhibit vesicular trafficking were identified, such as compound CLL-119 (IC_50_:14 µM) [50,51] (Figure 3). The complexity of the pentacyclic chiral scaffold of carpanone, polemannone, and sauchinone has not been an obstacle to the development of efficient approaches to this class of compounds. On the contrary, the chemical difficulty coupled with the multiple pharmacological properties of these compounds has stimulated the design and synthesis of analogues. Novel synthetic derivatives of sauchinone are regularly reported, such as the recently proposed *ent*-sauchinone-based amide derivatives (e.g., cpd 3 in Figure 3) with anticancer properties [52]. Sauchinone-type benzoxanthenones are attractive molecules but still underutilized in medicinal chemistry.

## 4. Metabolism of Sauchinone

Sauchinone exhibits favorable drug-like properties. An in silico analysis predicted drug-likeness with adherence to Lipinski’s rules of five and no toxic alert a priori. It is a small molecule (Mw = 356.13, 46 atoms), with a favorable water-octanol partition coefficient (logP = 2.978), a limited number of H-bond donor/acceptors (HBA = 6, HBD = 0), and a total polar surface area (TPSA) of 63.22 Å^2^. Calculations based on the PreADMET web server predicted that the compound exhibited an acceptable ADMET profile [53].

**Figure 4:**
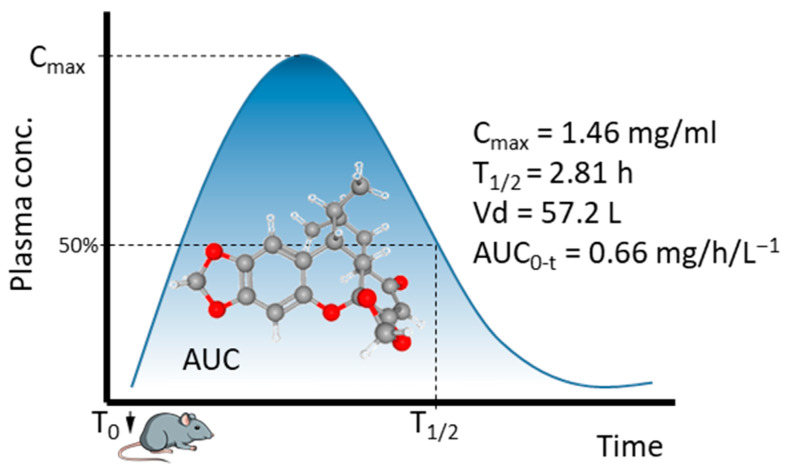
Pharmacokinetic parameters determined after a single intravenous administration of sauchinone at 10 mg/kg to Sprague–Dawley rats (C_max_, maximum plasma concentration; T_1/2_ = half-life; Vd, apparent volume of distribution; AUC_0–t_ = area under the plasma concentration time curve from 0 to t) [54].

Methods have been deployed to investigate the pharmacokinetic (PK) properties of sauchinone in animal models. Xu and coworkers developed a sensitive bioanalytical method using LC-MS (liquid chromatography with tandem mass spectrometric detection) for the quantification of sauchinone in rat plasma after an intravenous (iv) administration. A single-dose administration of the compound (10 mg/kg) to rats indicated that the molecule displays a good systemic exposure, with a maximum plasma concentration (C_max_) of 1.46 mg/mL and a half-life (T_1/2_) of 2.81 h (Figure 4). Apparently, the drug was well absorbed after a single-dose administration at 10 mg/kg (AUC_0−t_ = 0.66 mg/h/L^−1^) [54]. Sauchinone presented a linear PK upon iv administration to mice in the range 7.5–20 mg/kg. At a higher dose of 50 mg/kg, a non-linear PK was observed due to a metabolic saturation. When the compound was administered to mice orally at 20 mg/kg, 78% of the dose was absorbed, but the gastrointestinal absorption was considered to be moderate because the calculated absorbed fraction was low (F = 0.78 [suggesting that 7.8% of the orally administered dose is bioavailable]), possibly due to a significant hepatic and intestinal metabolism [55]. Different metabolites have been detected in the plasma, including mono- and di-oxidation products, mono- and di-methylation derivatives, dehydrogenation, and bis-glucuronidation of the natural product. The drug was found to be metabolized in the liver and small intestine, but also in extra-hepatic organs, and excreted into the urine predominantly via glomerular filtration [55,56].

In the liver, sauchinone is largely metabolized via interaction with uridine diphosphate (UDP)-glucuronosyltransferases (UGTs). The drug has been shown to inhibit primarily UGT2B7 and 1A6 (IC_50_ = 0.28 and 0.76 µM, respectively) and to a lower extent by UGT1A1 and 1A3 (IC_50_ = 8.83 and 43.9 µM, respectively) [57]. Another study reported the reversible and non-competitive inhibition of cytochrome P450s CYP3A4, 2B6, 2C19, 2E1, and 3A4 activities (with Ki values of 6.84, 14.3, 16.8, and 41.7 µM, respectively) when using human liver microsomes [58]. This type of inhibition can affect the metabolism of co-administered drugs. Therefore, caution should be exerted if sauchinone has to be combined with drugs primarily metabolized by the CYP isoform 3A4 and/or UGT isoform 2B7, such as zidovudine (azidothymidine, AZT) and others [57]. The inhibition can result in alteration in the PK parameters of the co-administered drug and may lead to drug-drug interactions.

## 5. Pharmacological Properties of Sauchinone and Congeners

Sauchinone and analogues have revealed multiple bioactivities, including antiviral, anticancer, antihypertensive, anti-inflammatory, immunosuppressive, hepatoprotective properties, and others. They are all mentioned below, with a focus on the antitumor properties of the compound.

### 5.1. Potential Antiviral Properties

The recent COVID-19 pandemic has stimulated the search for natural products susceptible to limit damages caused directly or indirectly by the severe acute respiratory syndrome coronavirus 2 (SARS-CoV-2) virus. In this context, sauchinone has been predicted to form a stable complex with SARS-CoV-2 main protease (Mpro) and to inhibit SARS-CoV-2 (Kd = 389.05 nM) [53,59]. Two other in silico studies have predicted binding of sauchinone to proteins of the SARS-CoV-2 virus. One modeling analysis defined the interaction of the drug with the Spike glycoprotein [60], and a docking study pointed out the formation of stable complexes with the 3-chymotrypsin-like cysteine protease (3CL(pro)) [61]. But at present, none of these in silico studies has been validated experimentally. Antiviral effects have been evidenced with extracts of the plant *S. chinensis*, but the activities were associated with the presence of lignans like manassantins A-B, saururin B, and saucerneols A-C, not sauchinone [62,63,64,65]. No direct effect of sauchinone on viruses, tropical parasites, fungi, and even bacteria has been reported. The compound presents an interest to combat bacterial infection via its capacity to increase macrophage phagocytosis, not via a direct antibacterial effect [66].

### 5.2. Protection from Organ Damages

**Figure 5:**
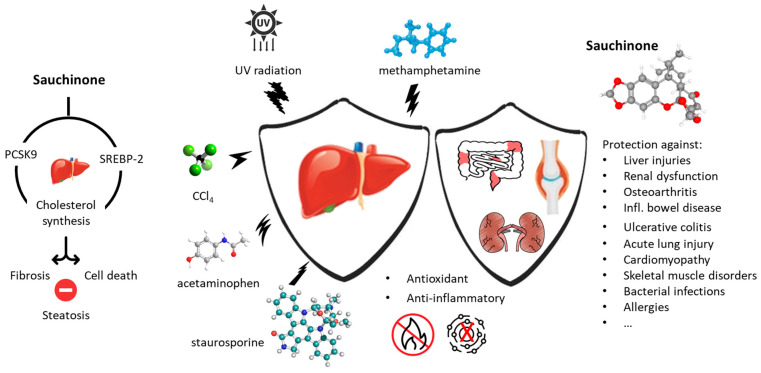
Organ-protective action of sauchinone against damages induced by diverse chemicals or radiations. The natural product exerts potent antioxidant and anti-inflammatory effects, which both contribute to protect damaged organs and tissues.

Sauchinone displays marked antioxidant and anti-inflammatory effects. It is a regulator of the oxidative defense system able to suppress pro-inflammatory mediators by inducing expression of heme oxygenase-1 (HO-1). This effect has been well evidenced in human umbilical vein endothelial cells (HUVEC) stimulated with high glucose. Sauchinone inhibited the phosphorylation and degradation of IκB-α and the nuclear translocation of transcription factor NF-κB p65 (nuclear factor-kappa B) caused by the stimulation of high glucose. At the same time, the compound induced HO-1 expression through translocation of the nuclear factor erythroid-2-related factor-2 (Nrf2) in HUVEC [67,68]. The capacity of sauchinone to upregulate HO-1 has been well demonstrated using different cell types, such as macrophages [68] and HepG2 liver cancer cells [69]. There are certainly different pathways leading to activation of Nrf2 by sauchinone because, in another context, the compound has been shown to trigger Nrf2 phosphorylation through activation of protein kinase C-δ (PKCδ) and an increased phosphorylation of glycogen synthase kinase-3β (GSK3β), which depressed Nrf2 activity. Through this mechanism, sauchinone was found to protect the liver from toxic effects of acetaminophen [70,71]. In fact, lignan serves as a protective agent against diverse chemicals, such as carbon tetrachloride (CCl_4_), methamphetamine, and staurosporine [72,73,74]. It protects also from damages induced by ultraviolet (UV) radiation [75] (Figure 5). The antioxidant effects of sauchinone have been largely reported using various experimental systems, with implications of multiple pathways depending on the cellular context [76,77]. With no doubt, sauchinone can serve as protector from oxidative stress, notably in the liver where the compound was found to induce fat accumulation and inhibition of cholesterol synthesis [78]. In HepG2 hepatocarcinoma cells, sauchinone showed a reduction in hepatic steatosis by downregulating the expression of hepatic PCSK9 (proprotein convertase subtilisin/kexin type 9) through protein SREBP-2 (sterol regulatory element binding protein-2), which is a transcriptional regulator of cholesterol homeostasis. Sauchinone prevents the synthesis of cholesterol via inhibiting SREBP-2 and its downstream targets [79]. As a PCSK9 inhibitor, sauchinone could be of interest to treat fatty liver disease [80]. The drug has been shown to attenuate liver fibrosis and other types of liver injuries [77,81,82]. The three isomers—sauchinone, sauchinone A, and 1′-*epi*-sauchinone—exhibit roughly the same hepatoprotective potency, with a capacity to block the release of glutamic pyruvic transaminase from CCl_4_-damaged hepatocytes in the range 70–78% [19].

The dual antioxidant and anti-inflammatory properties of sauchinone can be useful in other pathological states (Figure 5). For example, a study pointed out the capacity of the lignan to improve angiotensin II-induced renal fibrosis and inflammation, through reduction of the expression of Smad effectors [81]. The data suggested the interest of sauchinone to reduce renal damages in patients suffering from diabetic nephropathy [83]. Similarly, the drug was shown to inhibit angiotensin II-induced proliferation and migration of vascular smooth muscle cells, also through the TGFβ/Smad signaling pathway, thereby suggesting an interest for preventing vascular remodeling in hypertension [84]. There are many pathological conditions associated with an inflammation state and/or activation of oxidative pathways. The use of sauchinone could be useful to treat (i) osteoarthritis, via attenuation of the inflammatory response in chondrocytes and inhibition of osteoclast differentiation [85,86,87], (ii) inflammatory bowel disease (IBD), through reduction of intestinal inflammation and regulation of T cell functions [88,89,90], (iii) ulcerative colitis, via regulation of the NQO1/NF-κB pathway (NAD(P)H:quinone oxidoreductase 1) [91], (iv) acute lung injury, via attenuation of neutrophil pro-inflammatory activity and inhibition of TNFα expression in macrophages [92,93], (v) ischemia/reperfusion injury, via the protection of cardiomyocytes from cell death [94,95], (vi) airway inflammatory diseases such as allergic asthma and rhinitis, via the suppression of IL-4 production by Th2 cells and repression of Th2 cell development [96,97], (vii) viral-induced neuroinflammation, via inhibition of the expression of inducible nitric oxide synthase (iNOS) in microglial cells [98,99], (viii) skeletal muscle disorders, via the protection of myoblasts from oxidative stress [76], (ix) bacterial infection, through stimulation of macrophage phagocytosis [66], (x) attenuation of anxiety-like behavior in a model of ethanol withdrawal-induced anxiety, via inhibition of iNOS/nNOS protein expression (inducible/neuronal nitric oxide synthase) [100], and (xi) various types of allergies, via inhibition of bone marrow-derived mast cells (BMMC) and suppression of FcεRI-mediated mast cell activation [101]. The blockade of the production of pro-inflammatory mediators, such as nitric oxide (NO) and prostaglandin E2 (PGE2), by sauchinone can be useful in a large variety of pathological conditions. Several medicinal applications of sauchinone have been patented (Table 1).

**Figure 6:**
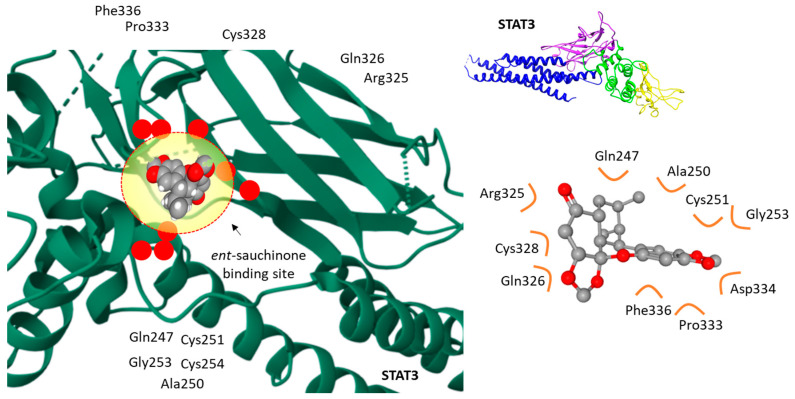
Molecular model of *ent*-sauchinone bound to STAT3 (PDB: 3CWG), adapted from Song and coworkers [102]. Through this protein interaction, the compound blocks the phosphorylation of STAT3, thereby inhibiting NF-κB activation and accumulation of amyloid Aβ plaques, implicated in Alzheimer’s disease.

The molecular target of sauchinone is not precisely known at present. A clever study has evidenced the direct binding of the derivative *ent*-sauchinone to protein STAT3 (signal transducer and activator of transcription 3) using a pull-down assay with an immobilized ligand (*ent*-sauchinone coupled to an epoxy-activated resin with cyanogen bromide). The direct binding of *ent*-sauchinone to STAT3 was evidenced, and a molecular modeling analysis supported the experimental observations. The compound can fit well into a concave binding pocket of STAT3, as represented in Figure 6. Via this process, the compound blocked the phosphorylation of STAT3 and inhibited STAT3-mediated NF-κB activity in cultured astrocytes and microglial BV-2 cells [102]. Those data suggested the interest in *ent*-sauchinone for the treatment of neuro-inflammatory diseases, including Alzheimer’s disease. The same compound *ent*-sauchinone has been shown to suppress STAT3 signaling in cancer cells, as discussed below. It is an inhibitor of STAT3 phosphorylation [103]. Similarly, the synthetic amide derivative of *ent*-sauchinone cpd 3 has been shown to bind to the DNA-binding domain of STAT3 and to inhibit phosphorylation of the transcription factor [52]. The same mechanism can be postulated with sauchinone. There is no data showing a direct binding of sauchinone to STAT3, but there is evidence for a repression of STAT activation by this product [99,104]. However, the point warrants further studies because there is also a report pointing out that sauchinone does not significantly affect the inhibition of STAT3 phosphorylation, but it could inhibit phosphorylation of other transcription factors such as CREB (cAMP response element-binding protein) or AP-1 (activator protein-1) [105,106]. The main molecular targets of sauchinone remain to be discovered.

### 5.3. Anticancer Activities

The anticancer activities of sauchinone have been evidenced with diverse types of cancer cells cultured in vitro and, in one case, using an in vivo model of cancer in mice. Early on, extracts of *S. chinensis* have been shown to trigger apoptosis of human prostate and breast cancer cells. Sauchinone was identified as a major active constituent of those extracts [107]. Over the past 15 years, sauchinone was shown to inhibit proliferation, and/or migration/invasion, or metastasis of a variety of cell types, including hepatocellular carcinoma cells [108], gastric cancer cells [109], lung adenocarcinoma cells [110], pancreatic adenocarcinoma cells [111], osteosarcoma cells [112], breast adenocarcinoma cells [105,113], colorectal carcinoma cells [114], and prostate adenocarcinoma cells [107,115]. In those studies, the drug was shown to restrict cell growth via the activation or suppression of several signaling pathways implicated either in cancer cell proliferation, migration, or invasion. For example, sauchinone-induced downregulation of epidermal growth factor receptor HER-2 has been evidenced using MCF-7 and Bcap-37 breast cancer cell lines [113], whereas inhibition of the phosphorylation of proteins Akt, ERK, and CREB, coupled to a suppression of the expression of matrix metalloproteinase (MMP)-13, was underlined when using the MDA-MB-231 and MTV/TM-011 breast cancer cell lines [105]. This latter study focused on MMP-13, whereas downregulation of the expression of MMP-2 and MMP-9 was evidenced when using SW480 and HCT116 colorectal cancer cells [114].

In lung adenocarcinoma, the main molecular pathway implicated in the drug action was a downregulation of eukaryotic translation initiation factor 4E-binding protein 1 (EIF4EBP1), known to play critical roles in the tumorigenesis and progression of solid tumors [110]. In liver cancer, the drug was shown to target the AMPK-mTOR pathway [108]. The compound exerts a direct action on cancer cells to limit their proliferation and movements, and in parallel, it may function as a regulator of immunity. An interesting study pointed out the downregulation of programmed death-ligand 1 (PD-L1) expressed in colorectal cancer cells [114]. Anticancer studies with sauchinone are relatively heterogeneous, but they all point out a capacity of the product to suppress tumor cell growth and migration/invasion in vitro. In particular, the compound was found to efficiently inhibit hypoxia-induced epithelial-mesenchymal transition (EMT). This important property has been evidenced in osteosarcoma cells, gastric cancer cells, and pancreatic adenocarcinoma cells [109,111,112]. A similar reversal of EMT has been observed recently with the aforementioned *ent*-sauchinone amide derivative 3 (Figure 3) targeting STAT3 [52]. Sauchinone regulates the oxygen defense system. The compound reduces oxygen deprivation and cell death via suppression of intracellular radical production. This mechanism is reminiscent of that observed in neuronal cells [78]. Sauchinone-induced suppression of the STAT3 signaling pathway has been invoked to explain the anti-migration/invasion capacity of the compound in liver cancer cells [103] and inhibition of neuroinflammation and amyloidogenesis in astrocytes and microglial BV-2 cells [102]. Therefore, the drug mechanism of action is not specific to cancer cells.

Preliminary proofs of activity in vivo have been reported recently. A modest inhibition of tumor growth was observed upon intravenous administration of sauchinone (5 mg/kg/day for 5 days) to mice bearing a xenografted Bcap-37 breast tumor [113]. But in vivo studies with sauchinone are rare, perhaps because of the limited oral bioavailability of the product. Further in vivo studies are needed to better appreciate the anticancer potency of the natural product. Thus far, all anticancer studies have been performed with sauchinone and derivatives (essentially in the *ent*-sauchinone series). There is a complete lack of biological information for the related products, carpanones and polemannones.

## 6. Conclusions

The natural products carpanone, polemannone, and sauchinone belong to a small group of benzoxanthenone lignans bearing a tetra, penta, or hexacyclic oxygenated skeleton. The first two natural products have been exploited essentially as chemical archetypes to synthesize derivatives. As a typical example, a large library of >10,000 molecules resembling carpanone, generated by solid-phase multicomponent reactions, has led to the identification of vesicular traffic inhibitors, such as derivative CLL-119 (IC_50_ = 13.9 µM) [50]. Beyond the chemistry efforts, carpanone and polemannone have been little studied at the pharmacological level. Polemannones A-C are the most neglected compounds in the series; their pharmacological properties have not been investigated despite the structural similarity with sauchinone.

Sauchinone has attracted considerable interest as an anti-inflammatory and antioxidant agent. It protects organs from oxidative damages via the suppression of intracellular radical production and regulates inflammation via the control of cytokine production. Notably, sauchinone is an efficient regulator of IL-1β-induced inflammatory response [85,86] and an inhibitor of NLRP3 inflammasome [83,116]. Its mechanism of action is complex, implicating several signaling pathways. The molecular targets are not well defined, but a few hypotheses have been raised, such as the binding of *ent*-sauchinone to STAT3 [102]. But more work is needed to characterize further potential protein targets for sauchinone and congeners.

The anticancer action of sauchinone has been amply documented using a variety of tumor cell lines. Here also, the mode of action appears to be heterogeneous, but the common denominator of these studies is an anti-migration and anti-invasion capacity. The compound markedly reduces the dynamic of cancer cells and also inhibits proliferation. But it is not a potent cytotoxic agent. It is rather a suppressor of cell invasion, of potential interest to reduce cancer cell metastasis. There is apparently no cell line selectivity: the drug inhibits migration of all types of solid tumor cells, such as breast, colon, gastric, and pancreatic cancer cells, to cite a few. New properties of sauchinone are emerging, such as the capacity to inhibit epithelial-mesenchymal transition and the regulation of cancer cell immunity, notably via inhibition of the PD-1/PD-L1 immune checkpoint [114]. The immune-mediated component of the mechanism of sauchinone is novel and promising. The immunomodulatory action of xanthenone anticancer agents has been known for a long time [117,118]. The immune action of sauchinone warrants further investigation. In addition, we are witnessing a renewed interest in sauchinone pharmacology, as reflected in recent publications and patents (Table 1) [116,119,120]. These novel aspects reinforce the interest in the compound and shall encourage the design of new synthetic analogues and derivatives. However, better evidence for an antitumor action in robust in vivo models is really needed. Hopefully, this review will encourage pharmacologists to further characterize the mode of action of sauchinone-type compounds.

## Figures and Tables

**Table 1:** Patents on sauchinone (or citing sauchinone) published between March 2025 and 2009.

Patent Numbers	Titles	Publication Dates (Owner)
CN119235730A	Composition with red fading effect and application thereof. Filed by Shanghai Jiakai Biological Co., Ltd. Published 2025-01-03.	2025-01-03 (Shanghai Jiakai Biological Co., Ltd.)
CN118845919A	A bacteriostatic, whitening, and tender composition containing *Impatiens balsamina* extract and its application.	2024-10-29 (Zhengzhou Yipin Pharmaceutical Technology Co., Ltd.)
CN118512378A	Whitening composition.	2024-08-20 (Plant Doctor Guangdong Biotechnology Co., Ltd.)
CN109828055A	A kind of method for building up of the HPLC fingerprint of *Saururus chinensis* medicinal material.	2022-03-22 (Guangdong Second Traditional Chinese Medicine Hospital. Sun Yat Sen University)
KR102138073B1	Composition comprising sauchinone for preventing or treating chronic pulmonary disease.	2020-07-27 (*)
CN106749311A	Reflect sauchinone analog derivative and its preparation method and application.	2019-03-29 (China Pharmaceutical University)
KR101887631B1	Composition for delaying senescence, lifespan extension, or preventing, treating, or improving diseases caused by lipofuscin accumulation comprising sauchinone as an active ingredient of *Saururus chinensis*.	2018-08-13 (*)
CN106810565B	Reflect sauchinone analog derivative and its application in antitumor drug is prepared.	2018-04-17 (China Pharmaceutical University)
CN103550370B	A kind of Chinese medicine compound for the treatment of gynecological inflammation.	2015-09-16 (Hunan Agricultural University)
KR101269208B1	Composition comprising sauchinone as an active ingredient for preventing or treating insulin resistance.	2013-05-30 (*)
CN103073560A	Sauchinone derivative and preparing method and application thereof.	2013-05-01 (Nanjing University of Chinese Medicine)
KR20130005118A	Pharmaceutical composition for preventing or treating muscle disease and disorders containing sauchinone.	2013-01-15 (*)
KR20090095309A	Composition for alleviation or treatment of atopic skin disease comprising sauchinone or *Saururus chinensis* (lour.) Baill extract having sauchinone.	2010-07-29 (*)
CN101372491A	A kind of enantiomer of lignan tribagudone ^1^ and its preparation method and liver protection application.	2009-02-25 (South China Sea Institute of Oceanology of CAS)

(*) Korean patents: authors cited in Korean language only. ^1^ Tribagudone is equivalent to *ent*-sauchinone.

## Data Availability

No new data were created.

## Abbreviations

The following abbreviations are used in this manuscript:

EIF4EBP1	eukaryotic translation initiation factor 4E-binding protein 1
EMT	epithelial-mesenchymal transition
GSK3β	glycogen synthase kinase-3β
HO-1	heme oxygenase-1
HUVEC	human umbilical vein endothelial cells
IBD	inflammatory bowel disease
IκB-α	inhibitor of kappaB alpha
MMP	matrix metalloproteinase
NF-κB	nuclear factor kappa B
Nrf2	nuclear factor E2-related factor 2
PCSK9	proprotein convertase subtilisin/kexin type 9
PD-L1	programmed death-ligand 1
PKCδ	protein kinase C-δ
rt	room temperature
SREBP-2	sterol regulatory element binding protein-2
STAT3	signal transducer and activator of transcription 3
